# Repair of the Peripheral Nerve—Remyelination that Works

**DOI:** 10.3390/brainsci3031182

**Published:** 2013-08-02

**Authors:** Åsa Fex Svennigsen, Lars B. Dahlin

**Affiliations:** 1 Institute of Molecular Medicine—Neurobiology Research, University of Southern Denmark, J.B. Winsløws vej 21.1, Odense DK-5000, Denmark; 2 Department of Clinical Sciences, Hand Surgery, Lund University, Skåne University Hospital, Malmö SE-20502, Sweden; E-Mail: Lars.Dahlin@med.lu.se

**Keywords:** peripheral nerve injury, Schwann cell, proliferation, bands of Büngner, nerve regeneration, myelination, target innervation, nerve repair

## Abstract

In this review we summarize the events known to occur after an injury in the peripheral nervous system. We have focused on the Schwann cells, as they are the most important cells for the repair process and facilitate axonal outgrowth. The environment created by this cell type is essential for the outcome of the repair process. The review starts with a description of the current state of knowledge about the initial events after injury, followed by Wallerian degeneration, and subsequent regeneration. The importance of surgical repair, carried out as soon as possible to increase the chances of a good outcome, is emphasized throughout the review. The review concludes by describing the target re-innervation, which today is one of the most serious problems for nerve regeneration. It is clear, compiling this data, that even though regeneration of the peripheral nervous system is possible, more research in this area is needed in order to perfect the outcome.

## 1. Introduction

A *peripheral nerve injury* (PNI) occurs when a nerve is compressed, crushed or severed and proper communication between the peripheral and central nervous system (PNS and CNS, respectively) is lost. PNI occurs in approximately 3% of all trauma patients and the incidence of a digital nerve injury requiring surgical nerve repair is 6.2/100000 inhabitants per year, making this type of injury significantly more common than e.g., spinal cord injuries, although the latter has a more severe impact on the individual [1,2,3]. Nerve compression lesions, such as carpal tunnel syndrome, has a prevalence of at least 3%–4%, where the condition is frequently surgically corrected with subsequent and recurrent sick leave; nerve injuries thus carry large societal costs. 

PNI is frequently located in the upper extremities and associated with a sub-optimal recovery of arm and hand function, loss of the capacity to move fingers and other joints, and sometimes a loss of sensation in the entire limb. The injuries often have severe consequences for the afflicted individuals, including loss of touch perception, impaired stereognosis, disturbed temperature perception, cold sensitivity, and although fortunately less frequently seen—pain, e.g., complex regional pain syndromes (e.g., CRPS 2) [4,5,6]. PNI leads to both individual suffering and altered/degraded quality of life for the patient [4,5,7]. Today, adult PNI patients may never achieve a useful degree of functional recovery. This is particularly evident for sensory recovery in adults, where, in spite of an adequate nerve repair, the brain is unable to adapt to and interpret new afferent signaling patterns from the periphery caused by misdirection of the axonal outgrowth after the nerve repair. Children, in contrast to adults, show an excellent clinical recovery after a PNI, which is explained by better regeneration as well as a superior plasticity of the young brain [1]. However, cerebral plasticity is beyond the scope of this review and the reader is referred to other reviews on this topic [2,6,8]. There are three other major reasons for an unsatisfactory rate of recovery in the peripheral nervous system: (1) lack of rapidly generated endogenous glial cells that can be used when performing an artificial bridging of a severed nerve; (2) no suitable, or insufficient amount of, materials to bridge a defect in the injured nerve, and; (3) clinical intervention at a time when the distal nerve segment has diminished or lost its responsiveness to outgrowing axons, e.g., the activation of Schwann cells is decreased over time after injury [9,10,11]. Neurons in the PNS have the potential to regenerate and reinnervate organs even after a severe PNI, but the potential for proper healing after available surgical treatments is dependent on factors distal to the injury. In this review, we describe molecular events from the moment of injury to when a natural healing process is underway, known primarily from research made in rodents. Relevant aspects related to the clinical practice of humans are discussed.

## 2. Initial Reaction to Injury at the Site of Lesion and in the Distal Nerve Segment

A PNI initiates a cascade of degenerative cellular and molecular changes at the site of injury. An influx of calcium into the Schwann cells occurs immediately after injury, as a result of mechanical insult and by the interruption of blood and oxygen supply [12,13,14,15,16]. Calcium stimulates early Schwann cell proliferation *in vitro* [17]. It also enters into the axoplasm of the injured axons, where it activates calpain, a protease essential for axonal degeneration [18,19]. The entrance of calcium into the axon at this point in time is also necessary for the formation of new growth cones [20,21]. A well-balanced concentration of Ca^2+^ may be essential for nerve regeneration, indicated by the fact that Ca^2+^ channel blockers may increase axonal outgrowth [4]. 

**Figure 1:**
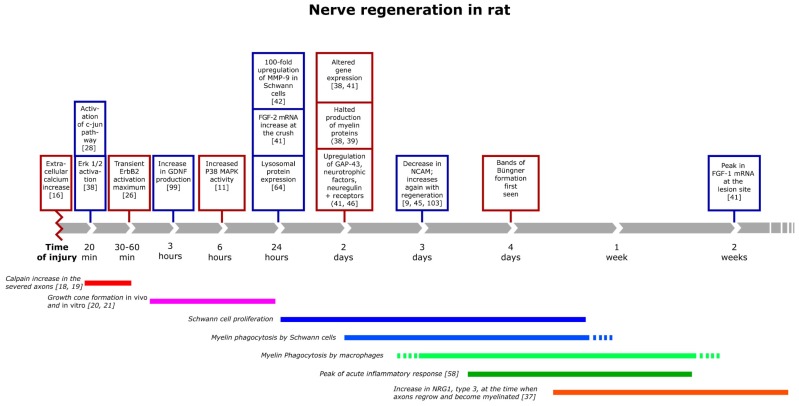
Timeline of major events important for nerve repair after injury, depicting many of the events known (that could be found in the literature) to occur after an injury. Most of these are likely important for the repair process.

The increase of calcium in turn activates intracellular cascades and gene regulatory proteins, such as mitogen-activated protein kinase family (MAPK), extracellular signal-regulated protein kinases (ERKs) and c-jun *N*-terminal protein kinases (JNKs) [22]. ERK 1/2 is activated already at 20 min post injury, while an activation of P38 MAPK appears six hours later (Figure 1). The activation of P38 MAPK, one of several molecules important for the progression of Wallerian degeneration, occurs subsequent to the increases in the levels of calcium, neuregulin, and Fibroblast Growth Factor-2 (FGF-2). This activation induces demyelination [23,24,25,26]. Further, down-stream in this signal cascade is the transcription factor c-jun, which is a global regulator of the Schwann cell injury response. The activation of c-jun is essential for the gene expression, for the function of the denervated Schwann cells and the formation of bands of Büngner and for Schwann cell proliferation and myelin clearance after injury in the distal segment (Figure 1) [11,27,28,29]. Without c-jun activation in Schwann cells, both unmyelinated and myelinated dorsal root ganglia (DRG) neurons are twice to three times as likely to die following axonal damage [29,30]. The absence of c-jun activation in Schwann cells also impairs axonal regeneration and results in a loss of the necessary increased expression of several neurotrophic factors that occur when c-jun is present [30]. The rapid activation of ERK 1/2 is a prerequisite for Schwann cell proliferation and the presence of ERK 1/2, as well as other transcription factors, like ATF 3, is important for axonal outgrowth [22]. These findings are particularly relevant since Schwann cell apoptosis increases significantly if nerve repair is delayed [31]. Even though these investigations are made in rodents, it likely applies to all mammals. It also underlines the importance of early repair after nerve injury in humans [9,11].

In peripheral nerve development, neuronal neuregulin I (NRG1) type III, present in axons, regulates nearly all stages of Schwann cell development. Moreover, axonal NRG1 type III determines myelin sheath thickness [32,33,34,35]. NRG1 is also necessary for degeneration, regeneration and subsequent remyelination in the PNS [36,37]. During the first one to three hours following nerve injury, phosphorylation of NRG1 receptors ErbB2 and ErbB3 are transiently up-regulated (Figure 1). This receptor activation indicates a transient availability of NRG1, possibly originating from the injured nerves [38]. Resident Schwann cells also increase their production of the secreted form of NRG1 type 1 shortly (one day) after injury [37]. The high concentrations of several of the NRG isoforms at the site of injury inhibits myelination, induces myelin degradation, Schwann cell proliferation and later Schwann cell migration [38,39,40]. 

Several other growth factors also increase at the site of injury during the first 24 h, including fibroblast growth factor 2 (FGF-2), which is up-regulated both distal and proximal to the injury and in the neurons in the DRG [41] (Figure 1). Metalloproteinase-9 (MMP-9) is found in adult nerves only after injury and predominantly in Schwann cells. MMP-9 belongs to a family of zinc endopeptidases that regulates the levels and the functionality of extracellular matrix components and cell surface signaling receptors [42]. After nerve damage, the production of MMP-9 increases over 100-fold in Schwann cells. MMP-9 activates critical trophic systems in Schwann cells and induces the Ras/Raf/MEK-ERK pathway via IGF-1, ErbB and Platelet derived growth factor (PDGF) tyrosine kinase receptors. MMP-9 may also be involved in Schwann cell mediated macrophage recruitment [43,44].

The net result of the early events of the nerve response to injury is Schwann cell proliferation, which in itself is an important event in the degeneration/regeneration process. Proliferation reaches a maximum two to three days after injury (Figure 1), and peaks approximately 24 h later in mixed nerves compared to unmyelinated nerves, due to the fact that myelin-forming cells need more time to adapt to their new role before they can proliferate [45,46,47]. The function of this injury-induced Schwann cell proliferation is likely to replace dead or dying cells, since Schwann cells are essential for the production of neurotrophic factors to support the survival of injured neurons, promote macrophage infiltration to the injured nerve and to supply growing axons with a substrate to grow on [48,49,50,51,52,53,54,55]. It is possible that Schwann cell proliferation is not as necessary as was previously anticipated for nerve regeneration, as mice, lacking the ability for Schwann cells to proliferate after injury, can regenerate normally in every respect compared to wild type mice [11,27]. Such an independence of regeneration from proliferation may indicate that Schwann cells acquire a regeneration-supportive phenotype without a need for cell division. Thus, Schwann cell proliferation may have other useful functions, such as building up the Schwann cell pool to meet the gradual death that occurs long term during regeneration of nerves in larger mammals.

## 3. The Inflammatory Response and Degradation of Myelin

Successful peripheral axonal regeneration is associated with a rapid and efficient inflammatory response [56]. The acute inflammatory response to PNI peaks four to seven days post injury, at the same time as the blood-nerve barrier becomes most permeable [56,57,58]. This allows blood-borne factors and cells that can facilitate tissue breakdown and repair to enter the nerve [56].

Toll-like receptors (TLRs) are known to be involved in the detection of microbial pathogens and activators of inflammatory response within cells [59]. TLRs have also been implicated in the recognition of tissue damage, such as nerve injury [60,61,62]. Schwann cells express several TLRs, some of which are up-regulated after injury [63]. Adding necrotic neurons, containing putative TLR ligands to cultured Schwann cells, also increases the expression of inflammatory mediators, such as tumor necrosis factor-alpha (TNF-α), inducible nitric oxide syntase (iNOS), and monocyte chemoattractant protein-1 (MCP-1) mRNA [64,65,66]. This indicates that endogenous TLR ligands, released from degenerating axons, bind TLRs on Schwann cells and immune cells, and activate inflammatory cascades, which likely are essential for promoting axon regeneration in mammals [56]. 

Schwann cells do not only degrade and break down their own myelin after injury, but also actively remove myelin debris and dead cells [67,68]. In fact, two days after injury most Schwann cells close to the site of injury contain degraded myelin sheaths, including “ovoids” (small myelin fragments), characteristic for Wallerian degeneration. At this early time point myelin-phagocytosis by macrophages is rarely detected. The phagocytosed myelin can then either be discharged and re-phagocytosed by macrophages or metabolized by the Schwann cells [69]. During myelin phagocytosis, Schwann cells in the distal stump also proliferate and secrete cytokines and chemokines that recruit immune cells to the injured nerve. The generation of proinflammatory cytokines, such as interleukin-6 (IL-6), leukocyte inhibitory factor (LIF), TNF-α, MCP-1, and interleukin-1α (IL-1α), starts within three to five hours of the injury in rodents [51,54,70,71,72]. 

The number of myelin-phagocytosing macrophages peaks one week after injury and consists of a mix of endogenous macrophages and those—the majority—that have been recruited from systemic circulation [69]. The proinflammatory cytokine IL-6 increases in both neurons and Schwann cells after injury and promotes neurite outgrowth [71,73]. Other neurotrophic factors, such as nerve growth factor (NGF), is also elevated at the site of injury and contributes to neuronal survival and re-growth [74]. NGF synthesis increases within hours of nerve injury and stays high for several weeks. Part of this prolonged increased synthesis is triggered by the release of IL-1 from macrophages invading the distal segment [75]. Excess macrophages remain in the nerve for days to months and finally either migrate to the lymphatic organs or die from apoptosis [76,77]. The T-lymphocytes are the last immune cells to arrive at a nerve injury and infiltrate the injured nerve after three days [78]. T-lymphocytes produce pro- and anti-inflammatory cytokines, which can either promote or inhibit macrophage function [56,79]. 

Thus, inflammation is vital for nerve regeneration to succeed. The cells most involved in the Wallerian degeneration, Schwann cells and macrophages, communicate via cytokine networks, controlling phagocytosis and growth factor release thus preparing the distal stump for axonal regeneration. In case of a delay in this degenerative/regenerative process, the cells lose their ability to promote regeneration. Future research will help determine how to manipulate the inflammatory responses to further improve repair and functional recovery.

## 4. Regeneration and the Relation between the Axon and the Schwann Cells

At the time when axons regenerate, the Schwann cells have started to proliferate, migrate and align within nerves the basal lamina tubes creating bands of Büngner and providing a guidance substrate for the re-growth of axons [80]. It has recently been suggested that NRG1, possibly in synergy with laminin, is responsible for Schwann cell migration in nerve regeneration [40,81]. The presence of laminin is also important for the outgrowth of axons in acellular nerve allografts, which are now clinically available to reconstruct nerve defects humans [82,83]. 

The Schwann cells of the bands of Büngner secrete growth factors and chemoattractants that stimulate and guide axonal growth. Several of these factors are neurotrophins, like brain derived neurotrophic factor (BDNF), and neurotrophic factors-3, -4/5, and -6 (NT-3, NT-4/5, and NT-6) [84]. Schwann cells, resident fibroblasts and growing axons all express the low affinity nerve growth factor receptor p75 receptor, which may play a role in the advancing of growth cones [85,86,87]. The Schwann cells in the bands of Büngner also produce an array of other growth promoting factors, such as insulin like growth factor-1 (IGF-1), ciliary neurotrophic factor (CNTF), glial derived neurotrophic factor (GDNF) and NRG1 [37,88,89,90]. All of these factors are by themselves, in synergy with each other, and especially with NGF, involved in axonal regeneration and in some cases Schwann cell proliferation both *in vivo* and *in vitro* [91,92,93,94,95,96,97,98,99]. Several adhesion molecules are also important for axonal guidance and are increased in the bands of Büngner. These are immunoglobulin (Ig)-like cell adhesion molecules (CAMs), like NCAM, cadherins, such as N-cadherin, and integrins [100,101]. The type of regenerating axon, *i.e.*, sensory or motor axon, influences expression of NCAM, which is expressed particularly by non-myelinating Schwann cells [9]. Interestingly, this adhesion protein is involved in the sorting of axons and Schwann cells and may perhaps preferentially promote the growth of sensory axons [9,102].

It is clear that several different growth factors and adhesion proteins affect both axonal sprouting and growth as well as Schwann cell proliferation and the formation of bands of Büngner. A better understanding of exactly which proteins are essential in promoting the regeneration process will be fundamental for future improvements in peripheral nerve surgery, like in timing of surgery and in development of new products to bridge nerve defects. 

## 5. Remyelination of Axons during and after Regeneration

As axons regenerate, the axon–Schwann cell interactions are renewed. This triggers remyelination and restoration of the physiologic function of the nerve fiber. Although regeneration and remyelination is possible in the PNS, the remyelinated axons often have thinner myelin sheaths and a decreased internodal length, leading to slower conduction [103]. This is observed even a long time (years) after nerve repair in human median and ulnar nerves and after decompression of the median nerve in carpal tunnel syndrome [27]. These unwanted results might be caused by insufficient stimulation of redifferentiated Schwann cells and/or by inhibitory signals. Schwann cells can also lose the ability to respond normally to myelination-inducing factors [37,103]. 

Recently, several investigations have shown that remyelination, as developmental myelination, may be dependent on neuregulin and that an addition of NRG1 improves the outcome of regeneration [37]. NRG1 type III increases in rodent motor neurons and DRGs two weeks after injury, the time of axonal regrowth and myelination [37,104]. Schwann cells also contribute to remyelination by expressing NRG1 type I at the site of injury [105]. NRG1 type I only increases if NRG1 type III is low, indicating that this autocrine or paracrine signal contributes to regeneration. Interestingly, neuronal NRG1 type III, decreases with age and may thus hamper axonal regeneration as well as remyelination in adult animals [34,36,106]. Furthermore, an increase of NRG1 types I and III, at the site of a nerve lesion, increases myelin thickness as well as the internodal length [36,37,81,106,107,108]. 

Many other molecules are also probably taking part in the remyelination process, even though their function is currently poorly understood. IGF-1, and its receptor, is up-regulated in Schwann cells in response to injury [109,110,111]. When Schwann cells are treated with IGF-1, *in vitro*, at the time of re-established axonal contact, axonal alignment, as well as the expression of myelinating genes, is enhanced [112]. IGF-1 is thus, similarly to NRG1, implicated in several different aspects of the regeneration process; Schwann cell proliferation, axonal growth, and myelination. 

Matrix metallopeptidase 9 (MMP-9) has recently been implicated in the myelination process as well as the determination of internodal length. In remyelinating mouse nerves lacking MMP-9, Schwann cells are unable to form long internodes. The inhibition of MMP-2/MMP-9 in myelinating DRG cultures also resulted in shorter internodes, implicating this protein in both developmental and regenerative generation of the nodes of Ranvier [113,114]. It is conceivable that endogenous NRG1, in combination with MMP-9, could help restoring nodes to their original internodal length after injury, and thus restoring proper conduction in remyelinated fibers. Other signals are also likely to be involved in this process. 

## 6. Target Innervation

Peripheral nerve injuries are rarely followed by complete return of function. In many peripheral nerves motor and sensory axons are intermixed and the correct choices for appropriate terminal nerve branches at the site of lesion are necessary for successful reinnervation of the right organ targets. Motor axons previously innervating muscle are often misdirected to sensory organs, and sensory axons typically innervating skin can be misdirected to muscle. The fact that different types of sensory axons reinnervate incorrect targets is a particular problem for the brain where the somatosensory cortex may have to interpret a new signal pattern from the periphery [2,6]. These phenomena are in fact a major barrier to functional recovery and outcome [115]. The distal nerve stump, as well as denervated target tissues, such as skeletal muscle, attracts axonal sprouts by providing chemotactic cues [116,117]. In rodents, and probably also in humans, the innervation of muscle is clearly more effective in comparison to skin- or sweat gland reinnervation [118]. 

The transcripts for BDNF, GDNF, and NT3 increase significantly in affected muscle after injury, while there is no such up-regulation in affected skin [118]. It is suggested that motor neurons assess the level of trophic support from each of the terminal branches and grow in the direction of the one that provides the greater amount of trophic support [115]. This is also consistent with classical work by Campenot showing that neurons retain their axons in compartments with relatively higher levels of trophic support [119]. Regenerating axons have the ability to distinguish between nerve and non-nerve targets, suggesting tissue specificity with respect to attracting and retaining regenerating axons [115]. Several of the growth factors important for regeneration and myelination are also essential for target innervation, The correct mix of factors to direct growing axons to either muscle or skin are not known, but different molecules likely stimulate sensory axons to skin and motor axons to innervate motor end plates in skeletal muscles. 

## 7. Conclusions

This review focuses on the Schwann cells and their fate during Wallerian degeneration, regeneration and the mechanism(s) behind remyelination. Similar and other, complementing, signal transduction pathways are also present in the neurons and are a prerequisite for initiating the regeneration process. Understanding of the behavior of the Schwann cells after injury is essential to further improve the results after treatment of peripheral nerve injuries. A deeper understanding concerning Schwann cells and their reactions, including a detailed description of the signal transduction pathways used in different steps of regeneration, may not only benefit patients suffering from PNI, but could potentially also help the development of new treatments for neuropathies common in several neurological diseases and after cancer treatment. In view of the global increase of subjects with diabetes, and the risk for complications such as diabetic neuropathy, the fate of Schwann cells is one target to improve the function and life of our patients. 

## Dedication

We dedicate the work on this paper to our friends and mentors Professor Martin Kanje, PhD, who died in March, 2013, and Dr. David R. Colman, PhD, who died in June, 2011. They have been an inspiration and a source of knowledge in our work with peripheral nerve regeneration and myelination.
